# Higher Ratio of Abdominal Subcutaneous to Visceral Adipose Tissue Related with Preservation of Islet *β*-Cell Function in Healthy Individuals

**DOI:** 10.1155/2017/6180904

**Published:** 2017-12-28

**Authors:** Juan Liu, Jianbin Liu, Hai Li, Liehua Liu, Jing Zheng, Zhimin Huang, Xiaopei Cao, Haipeng Xiao, Yanbing Li

**Affiliations:** ^1^Endocrinology Department, The First Affiliated Hospital of Sun Yat-sen University, Guangzhou 510080, China; ^2^Centre for Eye Research Australia, Faculty of Medicine, Dentistry and Health Science, The University of Melbourne, East Melbourne, VIC 3002, Australia; ^3^Box Hill Hospital Eastern Health, Box Hill, VIC 3128, Australia; ^4^Endocrinology Department, The Affiliated Hospital of Guizhou Medical University, Guangyang 550000, China

## Abstract

**Objective:**

To investigate the relationship between abdominal adipose tissue distribution, *β*-cell function, and insulin sensitivity (IS) in a Chinese population.

**Methods:**

One hundred and eighty-eight healthy subjects (healthy group), 239 with normal glucose, and 1~4 abnormal metabolic traits (metabolic dysfunction group, MD group) and 125 with hyperglycemia (hyperglycemia group) were studied. HOMA-IR, HOMA-B, Matsuda index, early- (I_0–30_/G_0–30_) and late-phase (I_30–120_/G_30–120_) insulin responses and the corresponding disposition indexes (DI) were calculated. The area of abdominal subcutaneous adipose tissue (ASAT) and visceral adipose tissue (VAT) was measured and the ratio of ASAT to VAT (SVR) was calculated.

**Results:**

SVR was correlated positively with Matsuda index in healthy, MD, and hyperglycemia groups, and inversely with HOMA-IR. SVR positively related with both early- and late-phase DI in the healthy group only. In the healthy group, the hyperbolas of I_0–30_/G_0–30_ and I_30–120_/G_30–120_ versus Matsuda index in the highest quarter of SVR were significantly right shifted compared to those in the lowest (both *P* < 0.05).

**Conclusions:**

In healthy adults, higher SVR was a protective factor for *β*-cell function and IS, while in those with glucometabolic abnormality, higher SVR contributed to a relative better IS, indicating SVR is possible to be an early predicator of type 2 diabetes development.

## 1. Introduction

In the last decade, China has experienced a substantial pandemic of diabetes and prediabetes, majority of which are type 2 diabetes mellitus (T2DM) [1]. It is of prime importance to identify individuals at risk of prediabetes and diabetes as early as possible, so that preventive strategies could be implemented at an earlier stage.

Impairment of islet *β*-cell function is the core determinant of the development of T2DM among the pathogenic factors and come out within the range of normal plasma glucose levels [2].

Compared to western populations, Asians tended to have worse *β*-cell function at the same body mass index (BMI) [3]. Asians also developed diabetes at younger ages, at lower degrees of obesity, and at higher rates given the same level of weight gain [4]. Interestingly, East Asians (such as Chinese, Japanese, and Koreans) have the most deleterious abdominal fat distribution across several ethnic groups, presenting the largest accumulation of visceral adipose tissue (VAT) but the lowest accumulation of deep subcutaneous adipose tissue (SAT) [5]. This “metabolically obese” phenotype among normal-weight individuals has been proposed as a major cause of the rapid increase in the prevalence of insulin resistance (IR) and T2DM in Asian populations. However, the mechanism of how this abdominal fat distribution pattern contributes to *β*-cell dysfunction is not yet clear.

Abdominal SAT (ASAT) and VAT have been shown to play different roles in the pathogenesis of T2DM and metabolic syndrome (MetS) [6–12]: ASAT can have beneficial effects on glucose metabolism [6–8] while VAT can increase IR. In studies with mice, decreases in body weight, fat mass, and blood glucose along with improved insulin sensitivity (IS) only occurred after SAT was transplanted to either visceral or subcutaneous regions [6]. In human studies, increased ASAT was negatively related to the prevalence of MetS [8], associated with decreased risk for IR, independent of VAT and BMI [9], while high VAT contributed to increased risks of MetS, T2DM, and even cardiovascular disease independent of BMI across races [10, 12].

The discrepant effect of ASAT and VAT on metabolism could be attributed to their differences in the type of adipocyte, endocrine function, lipolytic activity, insulin response, and so on [13]. Abdominal VAT is mainly distributed in the mesentery regions and the omentum and functions as a source of excessive nonesterified fatty acids (NFFA) which, via the portal vein system, deposit in undesirable sites such as liver and pancreas. Inflammatory meditators produced by adipocytes cause harmful effects on these organs and lead to IR or *β*-cell apoptosis [13]. On the contrary, the accumulation of SAT functions as a physiological buffer for excessive energy intake with limited energy expenditure, where the adipocytes act as a metabolic pool for excessive NFFA and glycerol to be stored in the form of triglycerides, thus exerting protective effects by reducing ectopic adipose tissue deposition [14].

Clinical studies found that abdominal adipose was associated with IR [9, 15, 16]; however, the relationship between ASAT or VAT and *β*-cell function in human seemed to be inconsistent [17–19]. Given that ASAT and VAT had opposite effects on metabolic profile, it is rational to take simultaneously both ASAT and VAT into consideration when assessing their effects on either *β*-cell function or IS. Therefore, we hypothesized that individuals with different abdominal adipose tissue distribution patterns (i.e., “more ASAT and less VAT” or “less ASAT and more VAT”) would have different *β*-cell function and IS status. The ratio of ASAT to VAT (SVR) was adopted to represent abdominal fat distribution. The correlation of SVR with the surrogate markers of *β*-cell and IS derived of oral glucose tolerance test (OGTT) was analyzed in a Chinese population with both normal and impaired glucometabolic status.

## 2. Methods

### 2.1. Subjects

The “Early Recognition and Intervention Technology Study of Metabolic Syndrome,” initiated by Shanghai Jiaotong University Affiliated Sixth People's Hospital, was a national multicenter cohort study conducted between December 2010 and December 2013 in China. This study included a baseline study and 1.5 and 3 yr follow-up. Subjects aged 40 to 65 years were eligible to enroll the study. The exclusion criteria were (1) known history of cardiovascular disease, (2) current treatment with systemic corticosteroids, (3) cirrhosis with or without ascites, (4) known hyperthyroidism or hypothyroidism, (5) severe disability and psychiatric disturbance, (6) presence of cancer, and (7) pregnancy. The study was approved by the Ethics Committee of Shanghai Jiaotong University Affiliated Sixth People's Hospital and written informed consent was obtained from each participant. As one of the collaborating centers in this study, the first affiliated hospital, Sun Yat-Sen University had enrolled a cohort in Guangzhou city. The current study was an analysis of baseline data of this cohort.

### 2.2. Body Composition and Measurements

Height and weight (both without shoes and in light clothing) were measured, and BMI (kg/m^2^) was calculated by dividing weight (kg) by height (m^2^) and waist circumferences (WC) measured at the midpoint between the lowest rib and the uppermost lateral border of right iliac crest. Systolic and diastolic blood pressure (SBP, DBP) was measured using a mercury sphygmomanometer at a resting state. Fat mass (FM, kg) and fat-free mass (FFM, kg) were measured by Tanita Bioelectrical Impedance MC-180 Analysis (Tanita Corp, Tokyo, Japan), with which FMI [FM (kg)/height (m^2^)] and FFMI [FFM (kg)/height (m^2^)] were calculated as previously described [20, 21].

### 2.3. Oral Glucose Tolerance Test (OGTT)

After 8–12 hours of overnight fasting, 75 g glucose solution was administered orally within 5 minutes. Blood samples were drawn before and at 30 min and 120 min postchallenge, and both glucose and insulin were measured. Fasting plasma glucose (FPG), triglycerides (TG), and high-density lipoprotein cholesterol (HDL) were measured using biochemical analysis.

Plasma glucose was determined by an enzymatic colorimetric test. Serum insulin levels were measured with an electrochemiluminescence immunoassay on a Cobas e411 analyzer (Roche Diagnostics GmbH, Mannheim Germany) with intra- and interassay coefficients of variation of 1.7% and 2.5%, respectively. TG was assayed by enzymatic colorimetric test with lipid clearing factor. HDL was measured enzymatically by direct method. The intra- and interassay coefficients of variation for lipid profile were less than 3% and 6%, respectively.

### 2.4. Measurement of Subcutaneous and Visceral Fat Using Magnetic Resonance Conventional T1 Weighted Imaging

Three plane localizers were scanned using a body coil. The central plane was placed through the navel, which was located using a vitamin E capsule, and the axial views were acquired. Six axial slices of T1-weighted images were obtained from all subjects [field of view (FOV) = 42 cm × 42 cm, slices thickness = 1 cm] with breath-holding, and the acquisition time was 12 s. For data analysis, the abdominal subcutaneous fat area (SFA) and visceral fat area (VFA) were measured to evaluate the abdominal subcutaneous adipose tissue (ASAT) and visceral adipose tissue (VAT). The measurement boundary for the region of interest (ROI) of the SFA was defined between the abdominal skin contour and the outer margin of the abdominal wall muscles, and the ROI of the VFA was delineated between the inner margin of the abdominal wall muscles and the anterior border of the spinal column. SLICE-O-MATIC version 4.2 software was used for imaging postprocessing. Taking the vertebral body signal as the reference standard, the software automatically identifies the pixels higher than the signal intensity of the vertebral body and automatically calculates the area. The same radiologist performed all of postprocessions.

### 2.5. Calculations

Homeostasis model assessment insulin resistance (HOMA-IR) was used to estimate hepatic insulin resistance, which was calculated as (FPG × fasting insulin (FINS))/22.5. HOMA of *β*-cell function (HOMA-B), calculated as 20 × FINS/(FPG − 3.5), was used to evaluate islet *β*-cell function [22].

Matsuda index, which provides an approximation of whole-body insulin sensitivity (IS), was defined as 10,000/([(FPG × fasting plasma insulin) × (mean glucose during OGTT × mean insulin during OGTT)])^1/2^ [23].

Basal disposition index (DI), a measure of secretory capacity of *β*-cell compensate for IR, was calculated as HOMA-B/HOMA-IR.

I_0–30_/G_0–30_, an index of early-phase insulin responsiveness after oral glucose challenge, is calculated as [[30 min insulin (INS) + FINS] × 30/2]/[(30 min plasma glucose (PG_30min_) + FPG) × 18 × 30/2] while I_30–120_/G_30–120_, an index of late-phase insulin responsiveness, defined as [(30 min INS + 120 min INS) × 90/2]/[(PG_30min_ + 120 min PG (PG_120min_) × 18 × 90/2)] [24]. Early-phase DI (I_0–30_/G_0–30_ × Matsuda index) and late-phase DI (I_30–120_/G_30–120_ × Matsuda index) were adopted to represent early- and late-phase insulin responsiveness to the oral glucose challenge with insulin sensitivity adjusted [24]. The glucose area under the curve (PG_AUC_) was calculated as the trapezoidal area during the 2-hour OGTT: [(FPG + PG30min)/2 × 30] + [(PG30min + PG120min)/2 × 90].

SVR, the ratio of ASAT to VAT, was defined as ASAT/VAT. Mean arterial pressure (MAP) was calculated by [(SBP × 2) + DBP]/3.

### 2.6. Determination of Metabolic Traits and Definitions of Subject Groups

The National Cholesterol Education Program/Adult Treatment Panel III (NCEP/ATP III) for Asian Americans [25] was adopted to define metabolic trait. The criteria were as follows:

1. Waist circumference ≥ 90 cm for men or ≥80 cm for women
2. Blood pressure ≥ 130/85 mmHg or currently using antihypertensive drugs
3. TG ≥ 1.7 mmol/l
4. HDL < 1.03 mmol/l for men or HDL <1.29 mmol/l for women

Meeting each of the above criteria was regarded as having one metabolic trait, and participants were divided into one of the three groups below:

- Healthy group: participants who had no metabolic trait (meeting none of the criteria) or hyperglycemia
- Metabolic Dysfunction (MD) group: participants with 1~4 metabolic traits (meeting 1~4 criteria) and normal blood glucose level of both FPG < 5.6 mmol/l and PG_120min_ < 7.8 mmol/l
- Hyperglycemia group: FPG ≥ 5.6 mmol/l or PG_120min_ ≥ 7.8 mmol/l or current use of medication(s) to treat hyperglycemia

### 2.7. Statistical Analysis

Statistical analysis was performed using SPSS18.0 software. Quantitative variables with normal distribution were presented as mean ± standard deviation (SD), whereas skewed variables as median (interquartile range). Skewed data underwent logarithmic transformation before statistical analysis.

Normally distributed variables and those following normal distribution after being log-transformed (HOMA-IR, Matsuda index, TG, I_0–30_/G_0–30,_ and early- and late-phase DI) were compared between the 3 groups by ANOVA with post hoc analysis, while those (FFMI, FMI, I_30–120_/G_30–120_, and basal DI) unable to be transformed to a normal distribution were compared using the Kruskal-Wallis H test among 3 groups and the Mann–Whitney *U* test between 2 groups with Bonferroni correction. Correlation coefficients were analyzed using Pearson's correlation (bivariate normal distribution satisfied) or Spearman's correlation (bivariate distribution violated).

To determine the independent association of SVR with *β*-cell function and IS, multivariate-adjusted regression model was performed with *β*-cell function and IS parameters as dependent variables and SVR as independent variable. All models were age and gender adjusted. Other body composition indexes such as BMI, ASAT, VAT, FMI, and FFMI were further introduced in model 2, and metabolic traits such as WC, MAP, HDL, TG, and FPG that have been reported as predicators of *β*-cell function and IS were stepwise introduced in model 3. In the group(s) in which the associations of SVR with *β*-cell function and IS are found to be statistically significant, participants were further divided into four groups according to quartiles of SVR. Glucose levels, *β*-cell function, and IS were compared among these four groups, using multivariate linear regression analysis to adjust for factors such as age, gender, and BMI. The hyperbolic relationships between I_0–30_/G_0–30_, I_30–120_/G_30–120_, and the Matsuda index were assessed by linear regressions of log I_0–30_/G_0–30_, log I_30–120_/G_30–120_, and the log Matsuda index. The slope of the regression line not significantly different from −1 indicates the hyperbolic relationship exists. If so, the positions of the hyperbolic lines in the four SVR quartiles were further compared by comparing the intercepts of the lines.

## 3. Results

In total, the study cohort comprised 552 individuals, of which 188 were in healthy group, 239 in MD group and 125 in hyperglycemia group.  Table 1 presents the demographic characteristics of the three groups as well as the parameters regarding glucose and lipid profiles, *β*-cell function, and IS along with abdominal adipose tissue distribution.

### 3.1. Comparison of Abdominal Fat Distribution and Glucose Metabolism among the 3 Groups (Table 1)

Compared to healthy group, the MD and hyperglycemic groups had higher ASAT and VAT. SVR was lower in MD group than that in healthy group and further declined in hyperglycemic group (All *P* < 0.05). Matsuda index had the same trend as observed in SVR while HOMA-IR trended in an opposite direction (all *P* < 0.05).

In regard to *β*-cell function, MD group had higher HOMA-B, I_0–30_/G_0–30_, and I_30–120_/G_30–120_ compared to the other two groups, while between healthy and hyperglycemic groups, statistically significant difference was observed only in HOMA-B. In comparison with those in healthy group, both early- and late-phase DI were lower in MD group and even more reduced in hyperglycemic group (all *P* < 0.05).

### 3.2. Correlation of Abdominal Fat Distribution with Glucose Metabolism

As shown in Table 2, both VAT and ASAT had positive correlation with HOMA-IR and negative correlation with Matsuda index in all groups. SVR was positively correlated with Matsuda index in all groups (*r* = 0.172, 0.159, and 0.237, resp., all *P* < 0.05) but inversely with HOMA-IR (*r* = −0.234, *P* < 0.01) only in hyperglycemic group. Both ASAT and VAT had positive correlation with all insulin secretion indexes in every group, but SVR had none.

Correlations of SVR with early- and late-phase DI were observed only in healthy group (*r* = 0.231 and 0.27, resp., both *P* < 0.05). VAT also correlated with both DIs in this group (*r* = −0.213, −0.218, both *P* < 0.05). Such correlations in MD and hyperglycemic group were of no statistical significance. ASAT showed no relationship with early- or late-phase DI in any group.

To assess whether SVR had independent correlation with glucose metabolism, multivariate-adjusted stepwise linear/logistic regression analysis was performed (Table 3). In healthy group, SVR was independently associated not only with early- and late-phase DI, but also with HOMA-IR and Matsuda index, and these associations slightly attenuated but remained statistically significant after the stepwise adjustment for age, gender, BMI, VAT, ASAT, LMI, FMI, and metabolic traits (Table 3). Whereas, in MD and hyperglycemic groups, SVR was related with HOMA-IR and Matsuda index but not with any DIs, irrespective of the adjustment.

### 3.3. Effect of SVR on the Hyperbolic Relationship of Insulin Response and Insulin Resistance in Healthy Group

Given that SVR was found to be independently correlated with early- and late-phase DIs in the healthy group only, we then performed linear regressions to test the hyperbolic relationship between insulin responsiveness and insulin sensitivity in this group of subjects. As revealed by linear regressions, both log I_0–30_/G_0–30_ and log I_30–120_/G_30–120_ were highly related with log Matsuda index (*r* = −0.729, −0.812, both *P* < 0.0001), and the slopes of the regression lines were not different from −1 (slope = −0.833 ± 0.182 and −0.837 ± 0.139, resp., both *P* > 0.05), which indicated the hyperbolic relationships were present.

**Figure 1:**
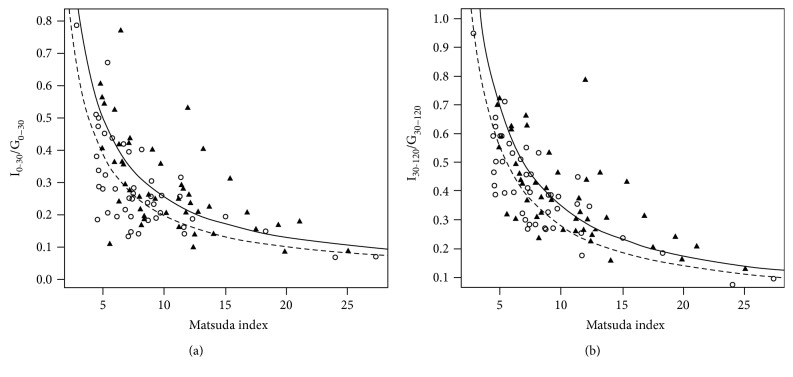
The hyperbolic relationship between I_0–30_/G_0–30_ and I_30–120_/G_30–120_ and Matsuda index in healthy group in which the healthy subjects were divided into four groups according to the quartiles of SVR. (a) The hyperbola of I_0–30_/G_0–30_ versus Matsuda index in quarter 4 (solid line and triangles, *intercept* = 0.435, regression *R* ^2^ = 0.401) was to the right of that in quarter 1 (dashed line and circles, *intercept* = 0.345, regression *R* ^2^ = 0.557); the lines of quarter 4 versus quarter 1: *t* statistic = 2.65, *P* = 0.01. (b) The hyperbola of I_30–120_/G_30–120_ versus Matsuda index in quarter 4 (solid line and triangles, *intercept* = 0.724, regression *R* ^2^ = 0.503) was to the right of that in quarter 1 (dashed line and circles, *intercept* = 0.848, regression *R* ^2^ = 0.721); the lines of quarter 4 versus quarter 1: *t* statistic = 2.91, *P* = 0.005.

Subjects in healthy group were further divided into four groups according to quartiles of SVR, characteristics of these four groups were presented in Supplementary Table 1. The positions of the hyperbolic curves of these four groups were compared by comparing of the intercepts of the curves. Only the difference between hyperbolas of quarter 4 and quarter 1 was statistically significant. As shown in Figures 1(a) and 1(b), the hyperbolas of quarter 4 were to the right of those of quarter 1 (both *P* < 0.05), indicating that the preservation of *β*-cell's secretory capacity during OGTT was better in subjects with high SVR in comparison with those with low SVR. And that, after adjusting for age, gender, and BMI, quarter 4 had a higher early- and late-phase DI, better IS, and even lower PG_30min_ than quarter 1 (seen in Supplementary Figures 1 and 2).

## 4. Discussion

In this study, we evaluated the effect of the abdominal fat distribution on *β*-cell function and IS by assessing the relationship of SVR (the ratio of ASAT to VAT) with the surrogate markers of *β*-cell function and IS derived from OGTT in a middle-aged Chinese cohort. A notable finding was that in metabolically normal individuals, SVR was related to insulin response after oral glucose challenge independent of BMI, VAT, and MetS components, and those with higher SVR had better insulin secretory capacity of *β*-cell (demonstrated as right shifted hyperbolas of I_0–30_/G_0–30_ and I_30–120_/G_30–120_ versus Matsuda index), indicating that higher subcutaneous with lower visceral fat accumulation would exert beneficial effect on *β*-cell function in the prevailing insulin sensitivity status. We also found higher SVR was associated with better IS which was consistent with previous studies [6, 7, 26], whereas in those with metabolic component(s) or hyperglycemia, higher SVR was strongly related with better IS but relationship between SVR and *β*-cell was insignificant.

It has been reported that *β*-cell function started to decline within the range of normal plasma glucose levels [2]. The mechanisms were unclear so far. Our study not only found similar *β*-cell function decline in healthy individuals but also revealed its relationship with abdominal adipose tissue distribution. According to our findings, it is rational to speculate that disarranged ASAT to VAT ratio could be a risk factor or even a trigger of *β*-cell function deterioration at a very early stage.

Most researchers studying how ASAT and VAT contributed to the pathophysiology of transition from obesity to T2DM [9, 15, 16] and their contribution in the pharmacological studies [11, 14] focused mainly the effects of ASAT and VAT on IR, while only a few assessed those on *β*-cell function, the results from which seemed to be inconsistent [17–19, 27]. Waist-to-hip ratio, an indicator that indirectly reflects abdominal fat distribution, was found to be an anthropometric modulator of *β*-cell function in healthy population [17]. Abdominal fat distribution was related with the difference in *β*-cell function among three ethnic races in Kenya [18]. However, in other studies, VAT was not associated with insulin secretion [19] and also lack of independent predictive effect on the incidence of T2DM [27]. In these studies, ASAT and VAT were not to be analyzed simultaneously in term of exploring their relationships with *β*-cell function, which may be partly attributable to the contrary results.

Recently, Gyllenhammer et al. [28] reported the findings in a longitudinal study of Hispanic children and young adults. They found that the ASAT was a predictor for insulin secretion, and over 2-year observation period, 1-SD increase in ASAT was significantly associated with a 55.6% increase in insulinogenic index (IGI), a 44.5% increase in *β*-cell function (calculated as the product of the ISI and IGI) and a 15.0% decrease in glucose AUC during OGTT, which were in agreement with our results.

The possible mechanisms by which high SVR confers beneficial effects on *β*-cell function are unclear, and the adipose tissue expandability hypothesis might provide some reasonable explanations. This hypothesis was trying to elucidate the mechanism of lipotoxicity at an individual level, which stated mainly that as a major fat storage site, ASAT had a defined limit of expansion for any given individual. If such limit was exceeded, net lipid flux to nonadipose organs would increase and lipids would begin to deposit ectopically. Ectopic lipid deposition in myocytes, hepatocytes, and *β*-cells then caused toxic effects such as IR and *β*-cells apoptosis [29]. It was probable that SAT determined whether ectopic lipid accumulation occurred and to which extent it could reach. So, the benefit of the increasing SAT on *β*-cell function, IR, and even glucose tolerance can be observed in rodent and human studies [6–10]. Conversely, functional SAT insufficiency has been discussed as a possible mechanism of failed adipocyte proliferation and differentiation [30]. In lipodystrophy, SAT dysfunction, characterized by paucity of subcutaneous fat, leads to markedly increased visceral and ectopic fat storage with severe IR and even hyperglycemia [31].

Interestingly, in this study, the benefit of high SVR for *β*-cell function was only observed in metabolically normal adults. The reason why the relationship between SVR and *β*-cell function was insignificant in other two groups was unclear. The participants in this study were classified into healthy, metabolic dysfunction, and hyperglycemic groups which could be regarded as representing the natural history of the development of type 2 diabetes across from insulin sensitive/normoinsulinemic, insulin-resistant/hyperinsulinemic, and insulin-resistant/hypoinsulinemic (or normoinsulinemic).

It is well recognized that IS is a major modulator of insulin secretion. The relationship between IS and insulin secretion is not linear and best described by a hyperbolic function [32]. The constancy of their product across a wide range of *β*-cell responses and IS is key to maintain normal glucose tolerance (NGT). In apparently healthy adults, *β*-cell responds appropriately and promptly to the varying IS, via augmenting or reducing insulin secretion to keep their product constant, reflecting a proper feedback loop regulating the interaction between *β*-cell and the peripheral tissues. So the influence of SVR on *β*-cell could be observed in these adults. However, once metabolic trait(s) and IR occurred, although these individual were still in NGT status, their *β*-cell functions had been impaired as revealed in our study and other studies [33, 34]. In such circumstance, the abovementioned feedback loop may be interrupted and the proportion of contribution of SVR on *β*-cell function may disappear or become much less and could not be detected by statistical method. In those with impaired glucose regulation, the damage of *β*-cell function became more evident, and at the same time more mechanisms such as glucotoxicity [35], lipotoxicity [36], inflammation [37], and oxidative stress [36] could emerge and together contribute to *β*-cell dysfunction, making the effect of SVR on *β*-cell negligible. In spite of this, high SVR still contributed to a relative better IS in these cohorts independent of hyperglycemia, dyslipidemia, hypertension, and abdominal obesity.

The strength of this study included the following: firstly, it was the first Chinese study in a relatively large population to evaluate the effect of abdominal adipose tissue distribution on *β*-cell function and IS. Secondly, ^1^H-MRS, an imaging technique regarded as the most reliable and validated method to assess abdominal adipose tissue area so far [38], was used to measure ASAT and VAT. Thirdly, parameters of *β*-cell function and IS were calculated from OGTT. These OGTT-derived estimates of insulin secretion correlated well with hyperglycemic clamp-derived measures [39] and provided various aspects of *β*-cell dynamics [24]. The limitations included the cross-sectional design of this study from which the causal association of SVR with *β*-cell function could not be evaluated. Liver and pancreatic fat volumes that might be also closely related with *β*-cell function and IS were not measured in this study, another limitation related to the generalizability to other populations. This study was conducted in Chinese subjects and would need to be confirmed in other populations.

## 5. Conclusion

In conclusion, in healthy adults, those with more ASAT and less VAT had better *β*-cell function and IS, while in those with metabolic dysfunction or hyperglycemia, high SVR was only associated with better IS, indicating abdominal SVR is possible to be a novel predicator of type 2 diabetes. Further studies assessing the causal relationship between ASAT and *β*-cell function are warranted.

## Figures and Tables

**Table 1:** The comparison of characteristics among the individuals in healthy, metabolic dysfunction (MD), and hyperglycemia group.

	Healthy group (*n* = 188)	MD group (*n* = 239)	Hyperglycemia group (*n* = 125)	*P* value
Demographics				
Patients (*n*)				
Male	68	98	58	
Female	120	141	67	
Age (years)	50.4 ± 6.5	50.8 ± 6.6	51.6 ± 7.1	0.352
Weight (kg)	56.6 ± 8.4	64.4 ± 10.2^∗^	64.5 ± 10.2^∗^	<0.0001
BMI (kg/m^2^)	21.7 ± 2.2	24.3 ± 2.8^∗^	24.5 ± 3.0^∗^	<0.0001
FMI (kg/m^2^)	5.8 (4.6–6.7)	7.1 (5.8–8.7)^∗^	6.9 (5.5–8.8)^∗^	<0.0001
FFMI (kg/m^2^)	15.4 (14.8–17.4)	16.4 (15.6–18.9)^∗^	16.7 (15.7–18.8)^∗^	<0.0001
Metabolic traits				
WC (cm)	74.3 ± 6.1	83.0 ± 8.0^∗^	83.4 ± 8.0^∗^	<0.0001
SBP (mm Hg)	107.9 ± 8.9	120.1 ± 14.9^∗^	119.4 ± 14.8^∗^	<0.0001
DBP (mm Hg)	71.6 ± 6.2	79.3 ± 10.1^∗^	78.3 ± 9.8^∗^	<0.0001
FPG (mmol/l)	4.9 ± 0.4	4.9 ± 0.4	5.7 ± 1.3^∗^	<0.0001
PG_30min_ (mmol/l)	7.8 ± 1.7	8.62 ± 1.9^∗^	10.6 ± 2.5^∗^	<0.0001
PG_120min_ (mmol/l)	5.2 ± 1.1	5.7 ± 1.2^∗^	10.0 ± 3.4^∗^	<0.0001
HDL (mmol/l)	1.67 ± 0.31	1.35 ± 0.33^∗^	1.35 ± 0.33^∗^	<0.0001
TG (mmol/l)	0.85 (0.70–1.10)	1.40 (0.90–1.90)	1.30 (1.00–2.00)	<0.0001
Abdominal fat distribution				
ASAT (cm^2^)	131.8 ± 46.5	176.7 ± 61.1^∗^	162.7 ± 59.9^∗^^#^	<0.0001
VAT (cm^2^)	45.2 (33.0–61.7)	84.3 (61.5–107.9)^∗^	90.3 (64.9–110.2)^∗^	<0.0001
SVR	2.8 (2.2-3.6)	1.98 (1.5–3.0)^∗^	1.8 (1.4–2.3)^∗^^#^	<0.0001
Insulin resistance/sensitivity				
HOMA-IR	1.05 (0.74–1.40)	1.61 (1.11–2.28)^∗^	2.15 (1.56–3.34)^∗^^#^	<0.0001
Matsuda index	8.8 (6.2–11.8)	5.8 (4.0–7.9)^∗^	4.1 (2.6–5.7) ^∗^^#^	<0.0001
Insulin secretion				
HOMA-B	75.2 (53.6–100.8)	107.8 (77.0–150.5)^∗^	95.5 (54.7–149.6)^∗^^#^	<0.0001
I_0–30_/G_0–30_	0.2 (0.2–0.4)	0.3 (0.2–0.5)^∗^	0.2 (0.2–0.4)^#^	<0.0001
I_30–120_/G_30–120_	0.4 (0.3–0.5)	0.5 (0.3–0.6)^∗^	0.4 (0.3–0.6)^#^	<0.0001
Disposition indexes				
Basal DI	72.1 (55.2–88.9)	65.6 (50.9–88.9)	47.2 (29.2–65.6)^∗^^#^	<0.0001
Early-phase DI	2.1 (1.7–2.8)	1.7 (1.4–2.3)^∗^	0.9 (0.6–1.2)^∗^^#^	<0.0001
Late-phase DI	3.1 (2.6–3.8)	2.6 (2.1–3.2)^∗^	1.6 (1.3–2.1)^∗^^#^	<0.0001

Normally distributed data are presented as mean ± SD. Nonnormally distributed data are presented as median (interquartile range). ^∗^Difference between healthy group versus metabolic dysfunction (MD) group: *P* < 0.05. ^#^Difference between MD group versus hyperglycemia group: *P* < 0.05. BMI: body mass index; FFMI: fat-free mass index; FMI: fat mass index; SBP: systolic blood pressure; DBP: diastolic blood pressure; FPG: fasting plasma glucose; PG_30min_: 30 min plasma glucose during oral glucose tolerance (OGTT); PG_120min_: 120 min plasma glucose during OGTT; TG: triglyceride; HDL: high-density lipoprotein; ASAT: abdominal subcutaneous adipose tissue; VAT: visceral adipose tissue; SVR: the ratio of SAT to VAT; HOMA-IR: homeostasis assessment insulin resistance; HOMA-B: HOMA *β*-cell function; Matsuda index was calculated as 10,000/([(FPG × fasting plasma insulin) × (mean glucose during OGTT × mean insulin during OGTT)]). Basal DI: basal disposition index (DI) was calculated as HOMA-B/HOMA-IR; I_0–30_/G_0–30_ was calculated as [[30 min insulin (INS) + FINS] × 30/2]/[(30 min plasma glucose (PG) + FPG) × 18 × 30/2]; early-phase DI was calculated as I_0–30_/G_0–30_ × Matsuda index. I_30–120_/G_30–120_ was calculated as [(30 min INS + 120 min INS) × 90/2]/[(30 min PG + 120 min PG) × 18 × 90/2]; late-phase DI was calculated as I_30–120_/G_30–120_ × Matsuda index.

**Table 2:** Correlations coefficients of SAT, VAT, and SVR with glucose metabolism.

	Healthy group (*n* = 188)			MD group (*n* = 239)			Hyperglycemic group (*n* = 125)		
	ASAT	VAT	SVR	ASAT	VAT	SVR	ASAT	VAT	SVR
Glucose profile									
FPG	0.101	0.148^∗^	−0.086	0.017	0.179^∗∗^	−0.139^∗^	0.066	0.100	−0.094
PG_30min_	0.09	0.193^∗^	−0.295^∗∗∗^	−0.092	0.303^∗∗^	−0.342^∗∗∗^	0.082	0.093	−0.037
PG_120min_	0.106	0.186^∗^	−0.104	0.001	0.051	−0.022	−0.032	0.137	−0.185^∗^
Insulin resistance/sensitivity									
HOMA-IR	0.325^∗∗∗^	0.369^∗∗∗^	−0.139	0.278^∗∗∗^	0.295^∗∗∗^	−0.053	0.307^∗∗^	0.459^∗∗∗^	−0.234^∗∗^
Matsuda index	−0.294^∗∗∗^	−0.384^∗∗∗^	0.172^∗^	−0.210^∗∗^	−0.361^∗∗∗^	0.159^∗^	−0.299^∗∗^	−0.467^∗∗∗^	0.237^∗∗^
Insulin secretion									
HOMA-B	0.274^∗∗∗^	0.266^∗∗∗^	−0.064	0.287^∗∗∗^	0.174^∗∗^	0.050	0.255^∗∗^	0.321^∗∗^	−0.092
I0–30/G0–30	0.303^∗∗∗^	0.204^∗^	0.031	0.216^∗∗^	0.260^∗∗∗^	−0.080	0.199^∗^	0.333^∗∗∗^	−0.145
I_30–120_/G_30–120_	0.311^∗∗^	0.275^∗∗^	−0.019	0.188^∗∗^	0.276^∗∗∗^	−0.104	0.255^∗∗^	0.320^∗∗∗^	−0.075
Disposition indexes									
Basal DI	−0.090	−0.147^∗^	0.091	−0.019	−0.176^∗∗^	0.137^∗^	−0.057	−0.097	0.097
Early-phase DI	0.011	−0.213^∗∗^	0.231^∗∗^	−0.006	−0.118	0.089	−0.098	−0.076	0.061
Late-phase DI	−0.045	−0.218^∗∗^	0.217^∗∗^	−0.079	−0.130	0.064	−0.081	−0.102	0.097

^∗^ *P* < 0.05; ^∗∗^ *P* < 0.01; ^∗∗∗^ *P* < 0.0001. ASAT: subcutaneous adipose tissue; VAT: visceral adipose tissue; SVR: the ratio of SAT to VAT; FPG: fasting plasma glucose; PG_30min_: 30 min plasma glucose during oral glucose tolerance (OGTT); PG_120min_: 120 min plasma glucose during OGTT; HOMA-IR: homeostasis assessment insulin resistance; HOMA-B: HOMA *β*-cell function; Matsuda index was calculated as 10,000/([(FPG × fasting plasma insulin) × (mean glucose during OGTT × mean insulin during OGTT)]). Basal DI: basal disposition index (DI) was calculated as HOMA-B/HOMA-IR; I_0–30_/G_0–30_ was calculated as [(30 min insulin (INS) + FINS) × 30/2]/[(30 min plasma glucose (PG) + FPG) × 18 × 30/2]; early-phase DI was calculated as I_0–30_/G_0–30_ × Matsuda index. I_30–120_/G_30–120_ was calculated as [(30 min INS + 120 min INS) × 90/2]/[(30 min PG + 120 min PG) × 18 × 90/2]; late-phase DI was calculated as I_30–120_/G_30–120_ × Matsuda index.

**Table 3:** The multivariate-adjusted association of SVR with glucose metabolism in the stepwise linear/logistic regression analysis.

Healthy group (*n* = 188)		MD group (*n* = 239)		Hyperglycemia group (*n* = 125)	
Beta/OR (95% CI)	*P* value	Beta (95% CI)	*P* value	Beta/OR (95% CI)	*P* value
Log HOMA-IR					
Model 1 −0.036 (−0.061, −0.010)	0.006	−0.021 (−0.047, 0.005)	0.110	−0.060 (−0.107, −0.012)	0.014
Model 2 −0.048 (−0.072, −0.024)	0.000	−0.040 (−0.067, −0.014)	0.003	−0.051 (−0.094, −0.008)	0.021
Model 3 0.002 (0.001, 0.003)	0.010	−0.027 (−0.049, −0.004)	0.021	−0.061 (−0.103, −0.020)	0.004
Log Matsuda index					
Model 1 0.033 (0.009, 0.057)	0.007	0.035 (0.011, 0.059)	0.005	0.064 (0.022, 0.107)	0.003
Model 2 0.050 (0.027, 0.073)	0.000	0.049 (0.024, 0.074)	0.000	0.054 (0.016, 0.092)	0.006
Model 3 0.040 (0.017, 0.062)	0.001	0.037 (0.015, 0.059)	0.001	0.040 (0.003, 0.078)	0.036
Log early phase DI					
Model 1 0.023 (0.003, 0.042)	0.025	0.012 (−0.006, 0.030)	0.197	0.014 (−0.035, 0.063)	0.661
Model 2 0.023 (0.004, 0.041)	0.017	0.012 (−0.006, 0.030)	0.197	0.014 (−0.035, 0.063)	0.661
Model 3 0.018 (0.001, 0.035)	0.038	0.006 (−0.009, 0.022)	0.416	0.008 (−0.019, 0.039)	0.501
Log late phase DI				High late-phase DI^a^	
Model 1 0.020 (0.006, 0.035)	0.007	0.008 (−0.008, 0.023)	0.332	1.013 (0.636, 1.614)	0.956
Model 2 0.020 (0.006, 0.035)	0.006	0.007 (−0.008, 0.023)	0.335	1.558 (0.522, 4.651)	0.427
Model 3 0.018 (0.005, 0.031)	0.008	0.002 (−0.010, 0.015)	0.688	1.426 (0.414, 4.909)	0.574
High basal DI^b^					
Model 1 1.269 (0.984, 1.636)	0.067	1.139 (0.902, 1.439)	0.273	1.081 (0.670, 1.743)	0.750
Model 2 1.372 (0.820, 2.298)	0.229	1.112 (0.772, 1.600)	0.569	0.651 (0.207, 2.042)	0.462
Model 3 1.352 (0.791, 2.310)	0.270	1.232 (0.841, 1.804)	0.284	0.775 (0.243, 2.470)	0.667

Model 1: age and gender were included; model 2: BMI, SAT, VAT, FFMI, and FMI were included on the basis of model 1; model 3: WC, MAP, HDL, TG, and FPG were included on the basis of model 2; ^a^high late-phase DI was defined as the upper quartile of late-phase DI; ^b^high basal DI was defined as the upper quartile of basal DI. MAP indicates mean arterial pressure and is calculated by [(SBP × 2) + DBP]/3; HOMA-IR: homeostasis model assessment insulin resistance; HOMA-B: HOMA *β*-cell function; Matsuda index was calculated as 10,000/([(FPG × fasting plasma insulin) × (mean glucose during OGTT × mean insulin during OGTT)]). Basal DI: basal disposition index (DI) was calculated as HOMA-B/HOMA-IR; I0–30/G0–30 was calculated as [[30 min insulin (INS) + FINS] × 30/2]/[(30 min plasma glucose (PG) + FPG) × 18 × 30/2]; early-phase DI was calculated as I0–30/G0–30 × Matsuda index. I_30–120_/G_30–120_ was calculated as [(30 min INS + 120 min INS) × 90/2]/[(30 min PG + 120 min PG) × 18 × 90/2]; late-phase DI was calculated as I_30–120_/G_30–120_ × Matsuda index.

## Supplementary Materials

Supplementary Materials Supplementary Table S1: the characteristics of the healthy group in different quarters of SVR. Supplementary Figure S1: the comparison of *β*-cell function and insulin resistance/sensitivity in healthy group in which the healthy subjects were divided into four groups according to the quartiles of SVR. The data was shown as median and 2.5th~97.5th. ^∗^ *P* < 0.05 when compared to quarter 1 in the multiple linear analysis with age, gender, and BMI as covariates. Supplementary Figure S2: the comparison of fasting plasma glucose (FPG), plasma glucose 30 min (PG_30min_), and plasma glucose 120 min (PG_120min_) in the healthy group in which the healthy subjects were divided into four groups according to the quartiles of SVR. The data was shown as median and 2.5th~97.5th. ^∗^ *P* < 0.05 when compared to quarter 1 in the multiple linear analysis with age, sex, and BMI as covariates.
