# Supplementary material for: Higher Ratio of Abdominal Subcutaneous to Visceral Adipose Tissue Related with Preservation of Islet β-Cell Function in Healthy Individuals
Source: Int J Endocrinol. 2017 Dec 28;2017:6180904. doi: 10.1155/2017/6180904 (PMC5763169; doi:10.1155/2017/6180904)
Supplement: Supplementary Materials — Supplementary Table S1: the characteristics of the healthy group in different quarters of SVR. Supplementary Figure S1: the comparison of β-cell function and insulin resistance/sensitivity in healthy group in which the healthy subjects were divided into four groups according to the quartiles of SVR. The data was shown as median and 2.5th~97.5th. ∗ P < 0.05 when compared to quarter 1 in the multiple linear analysis with age, gender, and BMI as covariates. Supplementary Figure S2: the comparison of fasting plasma glucose (FPG), plasma glucose 30 min (PG30min), and plasma glucose 120 min (PG120min) in the healthy group in which the healthy subjects were divided into four groups according to the quartiles of SVR. The data was shown as median and 2.5th~97.5th. ∗ P < 0.05 when compared to quarter 1 in the multiple linear analysis with age, sex, and BMI as covariates. [file 6180904.f1.docx]

Supplementary table S1: The characteristics of the healthy group in different quarters of SVR

|  | Quarter 1  (SVR 0.8~2.16) | Quarter 2  (SVR 2.16~2.76) | Quarter 3  (SVR 2.76~3.64) | Quarter 4  (SVR 3.64~8.8) | *P* value |
| --- | --- | --- | --- | --- | --- |
| Patients (n) | 47 | 47 | 47 | 47 |  |
| Male | 28 | 18 | 13 | 9 |  |
| Female | 19 | 29 | 34 | 38 |  |
| Age (Years) | 52.2±6.0 | 50.6±7.4 | 48.6±5.5 | 50.3±6.6 | 0.064 |
| BMI (kg/m^2^) | 22.4±2.4 | 21.0±1.8 | 21.5±2.3 | 22.0±1.9 | 0.007 |

*

*

*

*

*

*

*

Supplementary figure S1: the comparison of β-cell function and insulin resistance/sensitivity in Healthy group in which the healthy subjects were divided into four groups according to the quartiles of SVR. The data was shown as median and 2.5^th^~97.5^th^. *: *P*<0.05 when compared to Quarter 1 in the multiple linear analysis with age, gender and BMI as covariates.


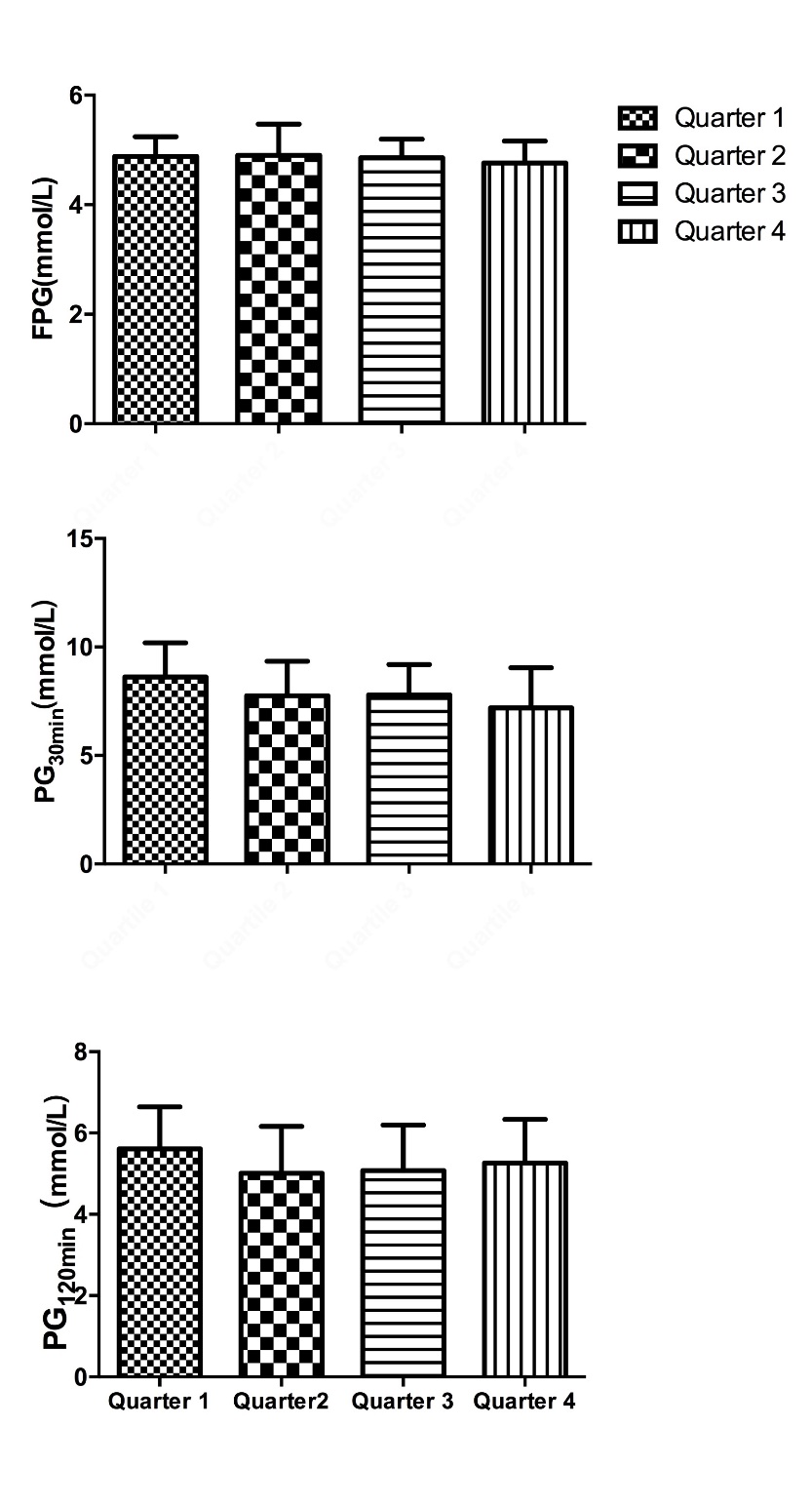


*

Supplementary figure S2: the comparison of fasting plasma glucose (FPG), plasma glucose 30 min (PG_30min_) and plasma glucose 120min (PG_120min_) in Healthy group in which the healthy subjects were divided into four groups according to the quartiles of SVR. The data was shown as median and 2.5^th^~97.5^th^. *: *P*<0.05 when compared to Quarter 1 in the multiple linear analysis with age, sex and BMI as covariates.
